# Long-lasting metabolic impairment in the failing heart: epigenetic memories at play

**DOI:** 10.1080/15592294.2025.2515430

**Published:** 2025-06-10

**Authors:** Sarah Costantino, Francesco Paneni

**Affiliations:** a Center for Translational and Experimental Cardiology (CTEC), Department of Cardiology, University Hospital Zurich and University of Zürich, Schlieren, Switzerland; b Department of Cardiology, University Heart Center, Cardiology, University Hospital Zurich, Zurich, Switzerland

**Keywords:** Heart failure, chromatin, epigenetics, LVAD, metabolism, mitochondria

## Abstract

Understanding the factors involved in myocardial recovery after unloading is of utmost importance to unveil new therapies in patients with heart failure (HF). Lack of myocardial recovery might be explained by long-lasting molecular alterations which persist despite normalization of cardiac stress. In this issue of Epigenetics, Roth et al. present an elegant translational study addressing this important aspect at the molecular level. By leveraging a mouse model of reversible transverse aortic constriction (rTAC) and human LV samples from HF patients undergoing LVAD therapy, the authors show that cardiac unloading is associated with a persistent deregulation of transcriptional programmes implicated in mitochondrial respiration, fatty acid and acyl-CoA metabolism, suggesting a long-lasting metabolic deterioration of the failing heart. Of interest, the authors identified several chromatin remodellers (Hdac4, Smarca2, and Brd4) potentially explaining the observed transcriptional alterations. Taken together, these novel findings suggest that ‘DNA forgives but does not forget,’ thus leaving an epigenetic scar which hampers the recovery of the failing heart after unloading. Disentangling the epigenetic factors involved in such ‘transcriptional memory’ may set the stage for new interventions resetting the cardiomyocyte transcriptome and myocardial energetics thus fostering a true myocardial recovery in HF.

Despite advances in medical treatment and device therapies, residual risk in patients with heart failure (HF) remains unacceptably high [1]. A medically important question which remains to be addressed is whether strategies fostering cardiac unloading may reverse functional and molecular features of the failing heart, thus leading to the so-called ‘true myocardial recovery’ and ‘myocardial remission’ [2]. Previous studies have shown that unloading of the human left ventricle by left ventricular assist device support (LVAD) could lead to myocardial recovery in selected cases [2,3]. However, data from the *Interagency Registry for Mechanically Assisted Circulatory Support* (INTERMACS) show that cardiac recovery during long-term LVAD therapy is reportedly as low as 1.3% [4]. In this context, addressing any reversible pathophysiologic processes that might impact reverse remodeling is important. Lack of myocardial recovery might be explained by long-lasting molecular alterations which persist despite normalization of cardiac stress. Other factors, including myocardial ischemia, use of cardiotoxic agents, arrhythmias, thyroid disease, and alcohol usage may also play an important role [3]. The identification of the mechanisms driving persistent myocardial damage after unloading is of outmost importance for the design of mechanism-based approaches accelerating myocardial recovery in these patients. This approach could also help identifying patients who have the ‘potential for recovery’ based on molecular landscape and functional features. In this issue of *Epigenetics*, Roth et al. [5] present an elegant translational study addressing this important aspect at the molecular level. By leveraging a mouse model of reversible transverse aortic constriction (rTAC) and human LV samples from HF patients undergoing LVAD therapy, the authors show that cessation of cardiac stress is not accompanied by a normalization or resetting of the myocardial transcriptome. Of interest, gene expression profiling in cardiomyocytes from rTAC mice unveiled a persistent deregulation of transcriptional programs implicated in mitochondrial respiration, fatty acid and acyl-CoA metabolism, suggesting a long-lasting metabolic deterioration of the failing heart despite cardiac unloading. Indeed, among the genes that were downregulated after TAC and did not recover, almost 40% encoded for mitochondrial proteins. Analysis of cardiac myocyte nuclei from non-failing, failing and LVAD-treated LV samples from HF patients confirmed a persistent deregulation of genes involved in aerobic respiration and acyl-CoA metabolism. Of note, these changes were associated with persistent upregulation of genes encoding for chromatin modifying enzymes namely *Hdac4*, *Smarca2*, and*Brd4* both in mouse and human cardiac myocytes. These findings help to understand the lack of a true myocardial recovery despite unloading. They also provide insights into potential epigenetic therapies able to rewire the cardiomyocyte transcriptome thus rescuing metabolic defects. The persistent upregulation of the epigenetic reader BRD4 is intriguing. This could be interpreted as a compensatory, yet futile mechanism to boost genes involved in mitochondrial metabolism, or, alternatively, as an active mechanism fostering BRD4-dependent genes involved in myocardial inflammation and fibrosis. Indeed, previous work has shown a strong involvement of BRD4 as a fine tuner of genes implicated in inflammation and fibrosis in experimental models of heart failure [6,7]. Whether BRD4 is friend or foe during cardiac unloading remains to be determined. Future studies in this direction could shed light on a potential target to restore cardiac energetics while suppressing lipotoxic injury. In line with these findings, previous work has shown that other forms of cardiac stress, namely hyperglycaemic stress in experimental diabetes, were linked to changes of the epigenetic landscape (chromatin remodelling, microRNAs deregulation) and persistent deregulation of genes involved in oxidative stress and inflammation [8,9].

In conclusion, the main message of this study is that ‘DNA may forgive but does not forget,’ thus leaving an epigenetic scar which hampers the recovery of the failing heart. Disentangling the epigenetic factors involved in such ‘transcriptional memory’ may set the stage for new interventions resetting the cardiomyocyte transcriptome and myocardial energetics thus fostering a true myocardial recovery.
